# Optimizing ceftolozane-tazobactam dosage during continuous renal replacement therapy: additional insights

**DOI:** 10.1186/s13054-019-2692-2

**Published:** 2019-12-12

**Authors:** Patrick M. Honore, Aude Mugisha, Leonel Barreto Gutierrez, Sebastien Redant, Keitiane Kaefer, Andrea Gallerani, David De Bels

**Affiliations:** ICU Department, Centre Hospitalier Universitaire Brugmann, Place Van Gehuchtenplein 4, 1020 Brussels, Belgium

We read the recent report by Aguilar et al., who concluded that among patients with nosocomial peritonitis who are on continuous renal replacement therapy (CRRT), ceftolozane-tazobactam (C/T) at a dose of 3 g every 8 h is safe [1]. This finding was additional information following the notion that CRRT was an independent predictor of clinical failure when C/T was administered at 1.5 g every 8 h [2]. The Aguilar et al. protocol included a short infusion time, i.e., 1 h [1]. Previously described extended-infusion over 4 h was found to reach above the minimal inhibitory concentration (MIC), given that beta-lactam antibiotics exhibit time-dependent antibacterial activity [3]. This might prevent underdosing during CRRT [3]. Besides, the C/T elimination was explained by diffusion [1]. However, adsorption was not assessed. The acrylonitrile 69 Multiflow (AN-69-M) membrane, used in this study, has a lower adsorptive capacity compared with the AN69 surface-treated (AN69-ST) membrane, which is considered a highly adsorptive membrane (HAM). In a recent comparison of polysulphone versus AN-69-M for C/T extraction by CRRT in an ex vivo model [4], there was no difference in adsorption. In a case report, a continuous infusion (CI) of 6 g in 24 h of C/T was used in a cystic fibrosis patient with a multidrug-resistant (MDR) *Pseudomonas aeruginosa* and augmented renal clearance to optimize time-dependent antibacterial activity [5]. In this patient, therapeutic drug monitoring (TDM) confirmed adequate exposure [5]. CI and TDM are two critical parameters when using C/T for patients receiving CRRT especially when MICs of bacteria like MDR *P. aeruginosa* are considered very high.

## Data Availability

Not applicable.

## Abbreviations

AN-69-M: Acrylonitrile 69 Multiflow
AN-69-ST: AN69-surface treated
C/T: Ceftolozane-tazobactam
CI: Continuous infusion
CRRT: Continuous renal replacement therapy
HAM: Highly adsorptive membranes
MDR: Multi-drug resistant
MIC: Minimal inhibitory concentration
